# Supplementary material for: Pre-trained molecular representations enable antimicrobial discovery
Source: Nat Commun. 2025 Apr 10;16:3420. doi: 10.1038/s41467-025-58804-4 (PMC11986102; doi:10.1038/s41467-025-58804-4)
Supplement: Supplementary file 1 — Supplementary Information [file 41467_2025_58804_MOESM1_ESM.pdf]

Supplementary Information

Roberto Olayo-Alarcon[1,2,*], Martin K. Amstalden[3], Annamaria Zannoni[6], Medina Bajramovic[1], Cynthia M. Sharma[6], Ana Rita Brochado[3,4,5], Mina Rezaei[1], and Christian L. Müller[1,2,*]

[1]Institute of Statistics, Ludwig-Maximilians-Universität München
[2]Institute of Computational Biology, Helmholtz Munich
[3]Department of Microbiology, Biocenter, Julius-Maximilians-Universität Würzburg
[4]Interfaculty Institute of Microbiology and Infection Medicine Tübingen (IMIT), University of Tübingen
[5]Cluster of Excellence 'Controlling Microbes to Fight Infections' (CMFI), University of Tübingen
[6]Department of Molecular Infection Biology II, Institute of Molecular Infection Biology (IMIB), Julius-Maximilians-Universität Würzburg
[*]Corresponding author

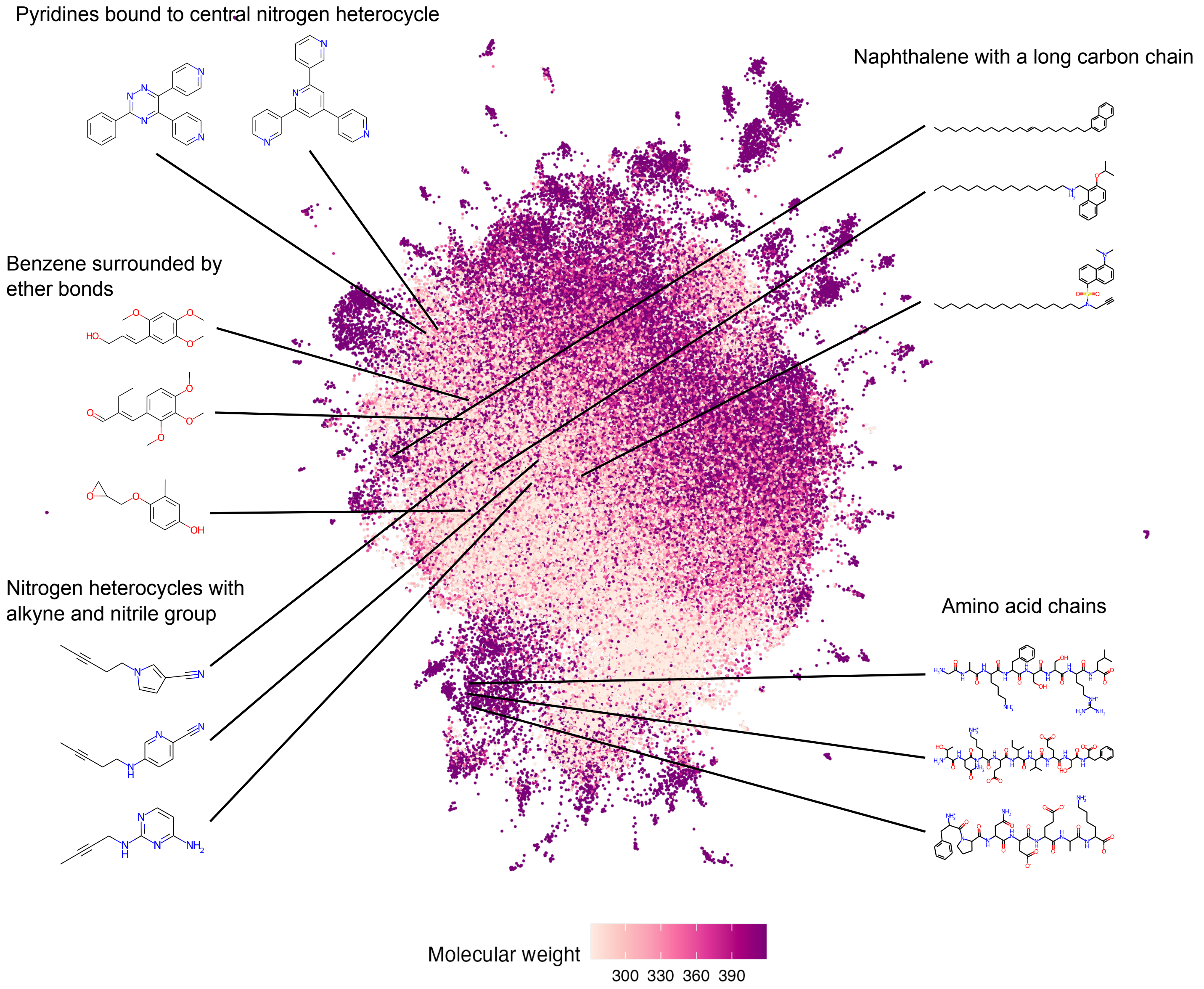

Supplementary Figure 1: UMAP embedding of the ECFP4 representation of 100,000 chemical structures not seen in pre-training. Compared to the MolE representation (Figure 2a in the main text), the ECFP4 representation is much less structured.

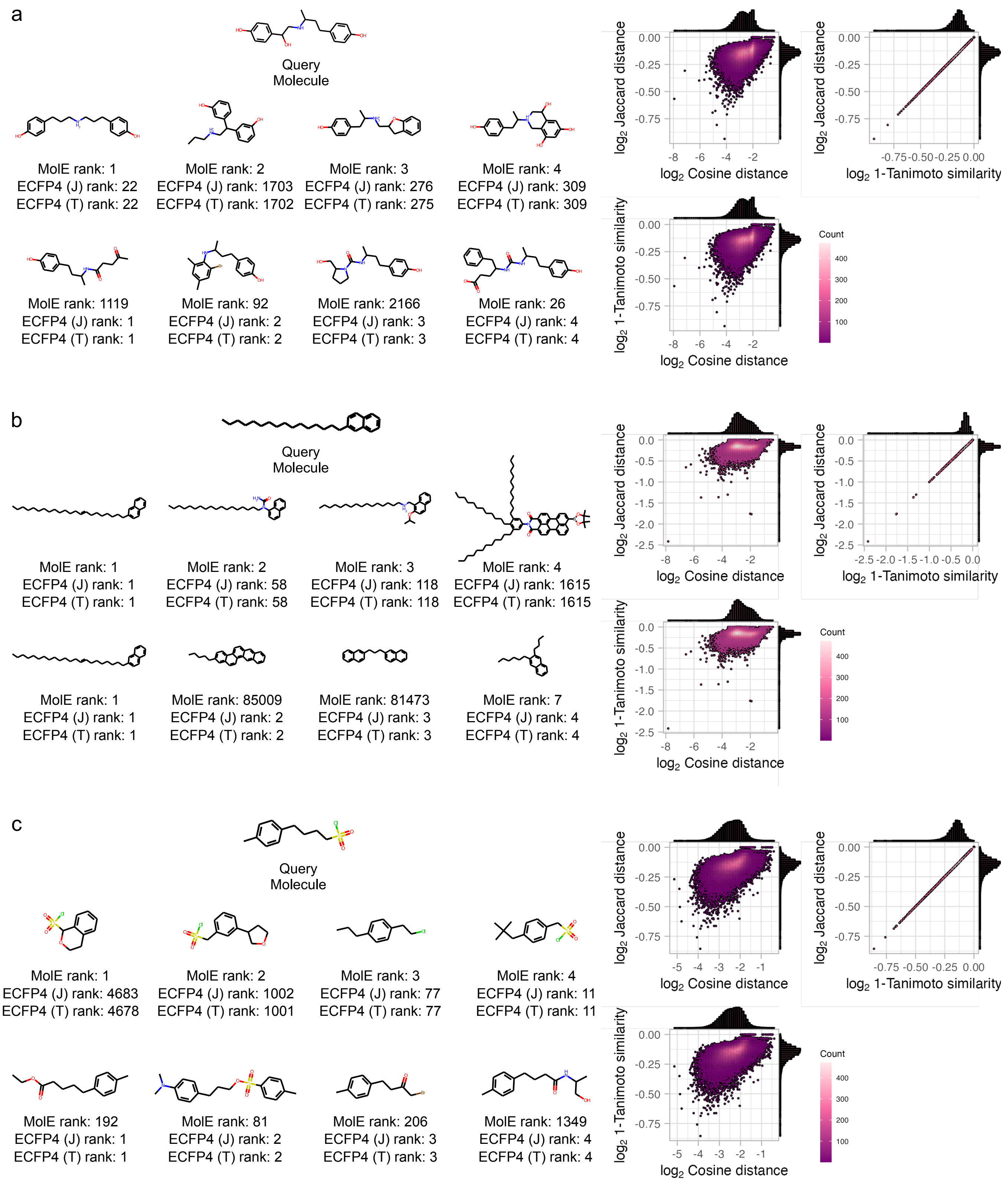

Supplementary Figure 2: Cosine distance is used to compare MolE representations, while the Jaccard (J) distance and Tanimoto (T) similarity are used for ECFP4. **a**. Molecular similarity to Ractopamine (PubChem ID: 56052). Top 4 most similar molecules according to MolE and ECFP4 (right). Distance with respect to all other molecules in the search space (left). **b**. Molecular similarity ranking with PubChem ID: 12277389 as query. **c**. Molecular similarity ranking with PubChem ID: 98701517 as query.

Performance on Regression Task
(Lower is better)

Dataset	FreeSolv	ESOL	Lipo	QM7	QM8
# Molecules	642	1128	4200	6830	21786
# Tasks	1	1	1	1	12
GCN	2.87±0.14	1.43±0.05	0.85±0.08	122.7±2.2	0.036±0.001
GIN	2.76±0.18	1.45±0.02	0.85±0.07	124.8±0.7	0.037±0.001
SchNet	3.22±0.76	1.05±0.06	0.91±0.10	**74.2±6.0**	0.020±0.002
MGCN	3.35±0.01	1.27±0.15	1.11±0.04	77.6±4.7	0.022±0.002
D-MPNN	**2.18±0.91**	**0.98±0.26**	**0.65±0.05**	105.8±13.2	**0.014±0.002**
ECFP4	4.08±0.00	1.58±0.02	0.92±0.00	169.85±2.81	0.024±0.000
N-Gram	2.99±0.00	**0.90±0.04**	0.78±0.00	102±2.45	0.027±0.000
$\text{MolCLR}_{\text{static}}$	3.65±0.06	1.43±0.02	0.90±0.02	203.41±0.00	0.030±0.000
$\text{MolE}_{\text{static}}$	**2.78±0.09**	0.95±0.04	**0.73±0.01**	**94.16±6.77**	**0.021±0.000**
Hu et.al	2.83±0.12	1.22±0.02	0.74±0.00	110.2±6.4	0.019±0.000
HiMol	**2.28±0.0**	**0.83±0.0**	**0.70±0.00**	91.5±0.0	**0.018± 0.00**
$\text{MolCLR}_{\text{finetune}}$	3.30±0.12	1.30±0.04	0.74±0.02	69.39±2.00	**0.018±0.004**
$\text{MolE}_{\text{finetune}}$	2.31±0.10	0.90±0.03	**0.70±0.02**	**58.19±1.56**	**0.018±0.000**

Supplementary Table 1: Average performance and standard deviation obtained on regression benchmark tasks. The first 5 models are supervised learning methods. The next 4 are the names of molecular features given as input to an XGBoost regressor. The final four methods are fine-tuned models. RMSE is shown for FreeSolv, ESOL, and Lipo. MAE is shown for QM7 and QM8. The best performance metric for each category is marked in **bold**.

Performance on Classification Tasks with Random Forest
(Higher is better)

Dataset	BBBP	Tox21	ClinTox	BACE	SIDER	HIV
# Molecules	2039	7831	1478	1513	1427	41127
# Tasks	1	12	2	1	27	1
ECFP4	72.29 ± 1.36	68.67 ± 1.48	76.86 ± 2.66	**84.53 ± 1.17**	62.69 ± 2.38	76.86 ± 1.28
N-Gram	73.31 ± 0.36	73.96 ± 1.48	**82.37 ± 3.5**	80.58 ± 0.92	65.59 ± 2.13	75.52 ± 1.01
$\text{MolCLR}_{\text{static}}$	69.01 ± 0.89	71.04 ± 1.93	76.32 ± 2.60	75.30 ± 1.96	64.12 ± 2.23	72.63 ± 1.09
$\text{MolE}_{\text{static}}$	**74.52 ± 1.24**	**75.98 ± 1.36**	78.57 ± 7.10	**84.60 ± 1.03**	**66.01 ± 2.49**	**78.34 ± 1.87**

Supplementary Table 2: Average ROC-AUC (%) and standard deviation obtained on classification benchmark tasks. The best performance metric is marked in **bold**

Performance on Regression Tasks with Random Forest
(Lower is better)

Dataset	FreeSolv	ESOL	Lipo	QM7	QM8
# Molecules	642	1128	4200	6830	21786
# Tasks	1	1	1	1	12
ECFP4	4.23±0.00	1.66±0.00	0.95±0.001	147.16±0.73	0.024±0.000
N-Gram	2.89±0.02	1.13±0.00	0.82±0.001	95.35±0.60	0.029±0.000
$\text{MolCLR}_{\text{static}}$	3.55±0.08	1.44±0.01	0.92±0.001	127.63±1.08	0.028±0.000
$\text{MolE}_{\text{static}}$	**2.80 ± 0.02**	**1.07 ± 0.01**	**0.79 ± 0.002**	**69.08 ± 0.11**	**0.022 ± 0.000**

Supplementary Table 3: Average performance and standard deviation obtained on regression benchmark tasks. RMSE is shown for FreeSolv, ESOL, and Lipo. MAE is shown for QM7 and QM8. The best performance metric is marked in **bold**

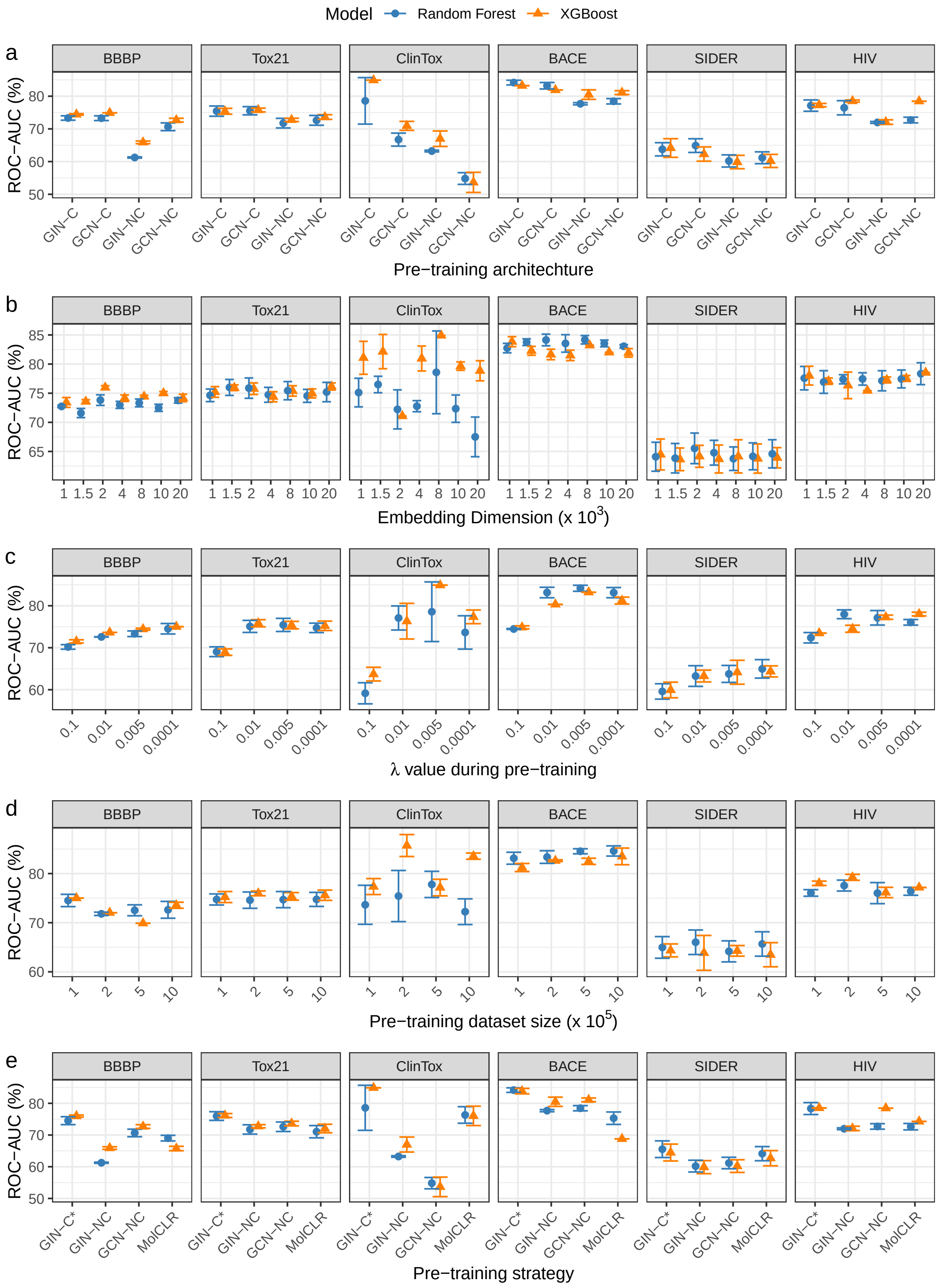

Supplementary Figure 3: Pre-training ablation study. Performance of classifiers when trained with representations from model ablations Average test-set ROC-AUC% from 3 training rounds (± standard deviation) show. **a**. GINs and GCNs are evaluated as GNN backbones in combination with representation vectors (**r**) built either by concatenating the update learned by each GNN layer (C) or by using the final GNN layer, with no concatenation (NC). **b**. Dimensionality of embedding vector (**z**). **c**. Different values of λ for the barlow-twins objective. **d**. Different pre-training dataset sizes. **e**. Performance of Random Forest/XGBoost models on pretrained GIN (concatenated and non-concatenated), GCN (non-concatended), and MolCLR (pretrained in the original publication) representations.

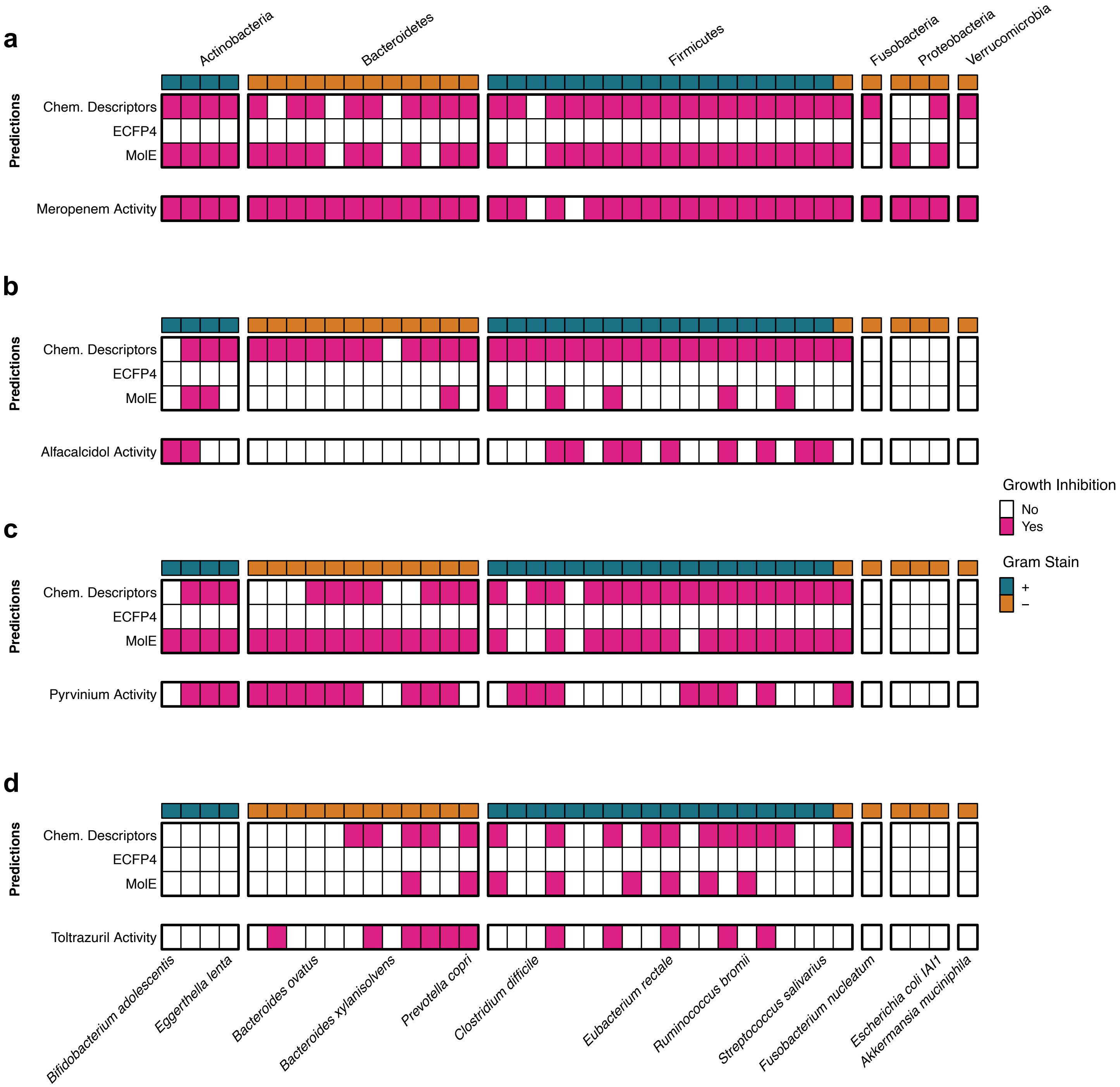

Supplementary Figure 4: Examples of molecules in the test set that are missed by ECFP4 and recovered by MolE. Every panel shows the predictions made by models trained with chemical descriptors, ECFP4 and MolE are show in the first three rows, while the ground truth is shown in the bottom row. **a**. Meropenem, is a known antibiotic. **b**. Alfacalcidol is a vitamin D analog. **c**. Pyrvinium is an anti-parasitic medication. **d**. Toltrazuril is an antiparsitic drug.

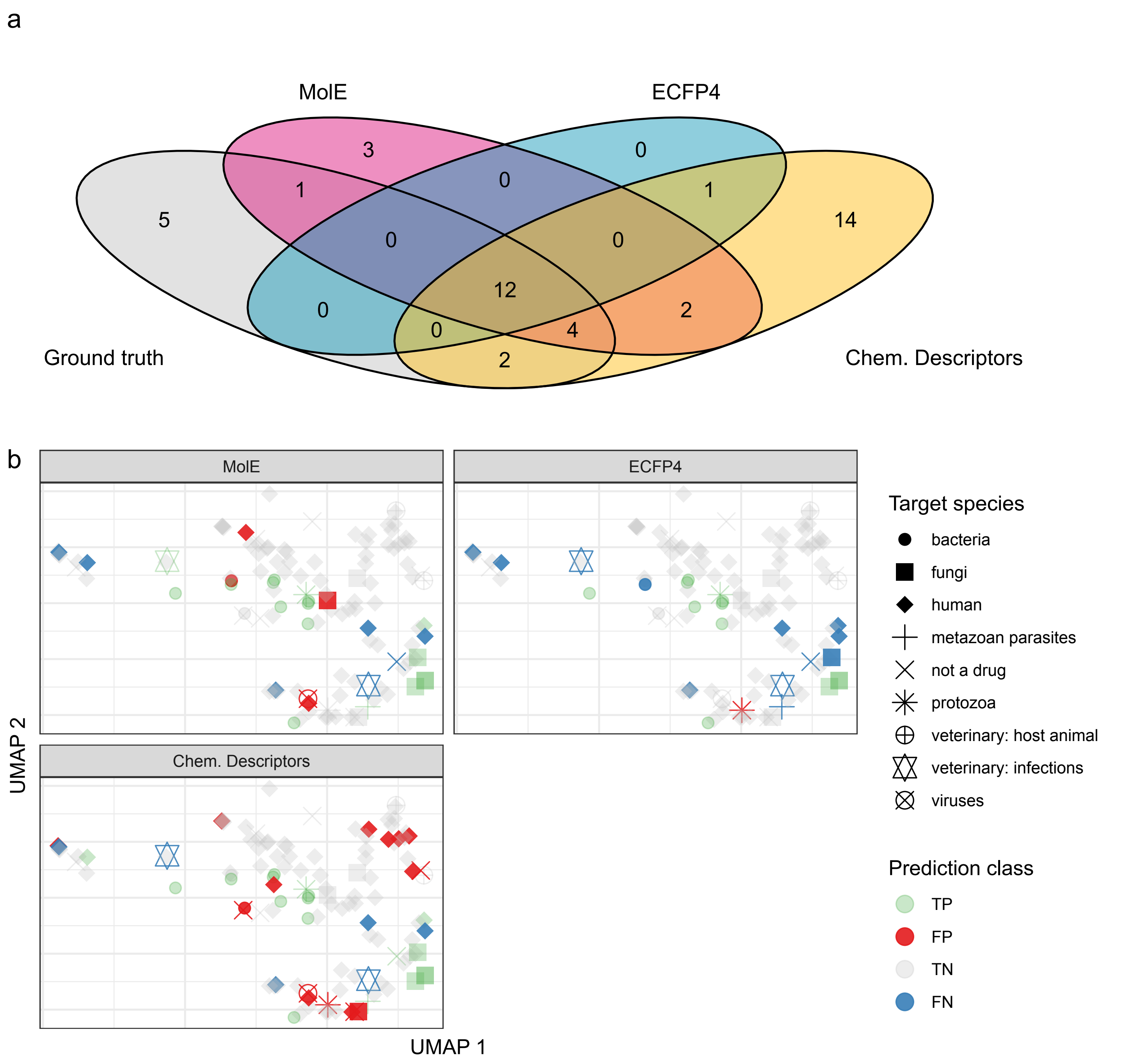

Supplementary Figure 5: Comparison of predicted broad-spectrum antimicrobial compounds in test-set. **a**. Overlap of compounds predicted to have broad-spectrum activity between the three models and the ground truth. **b**. UMAP embedding of MolE's representation of test-set molecules. Predictions of broad-spectrum activity are shown as True positive (TP), False positive (FP), False negative (FN), or True negative (TN). Each compound's intended target species is also shown.

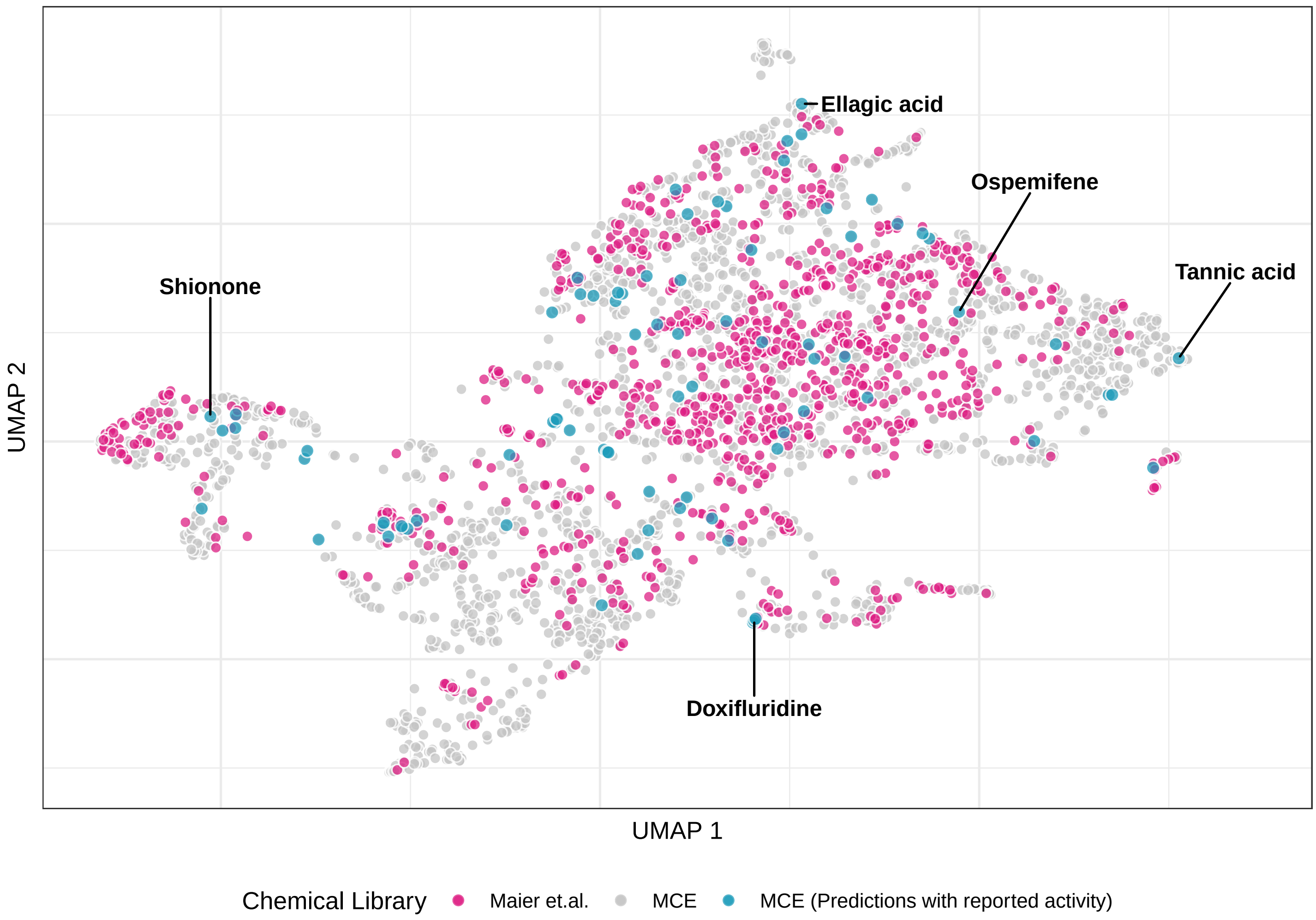

Supplementary Figure 6: A UMAP embedding of the MolE representation of the compounds in the MedChemExpress (MCE) and Maier et. al. libraries. Some examples of literature-validated predictions of antimicrobial compounds from MCE library are highlighted.

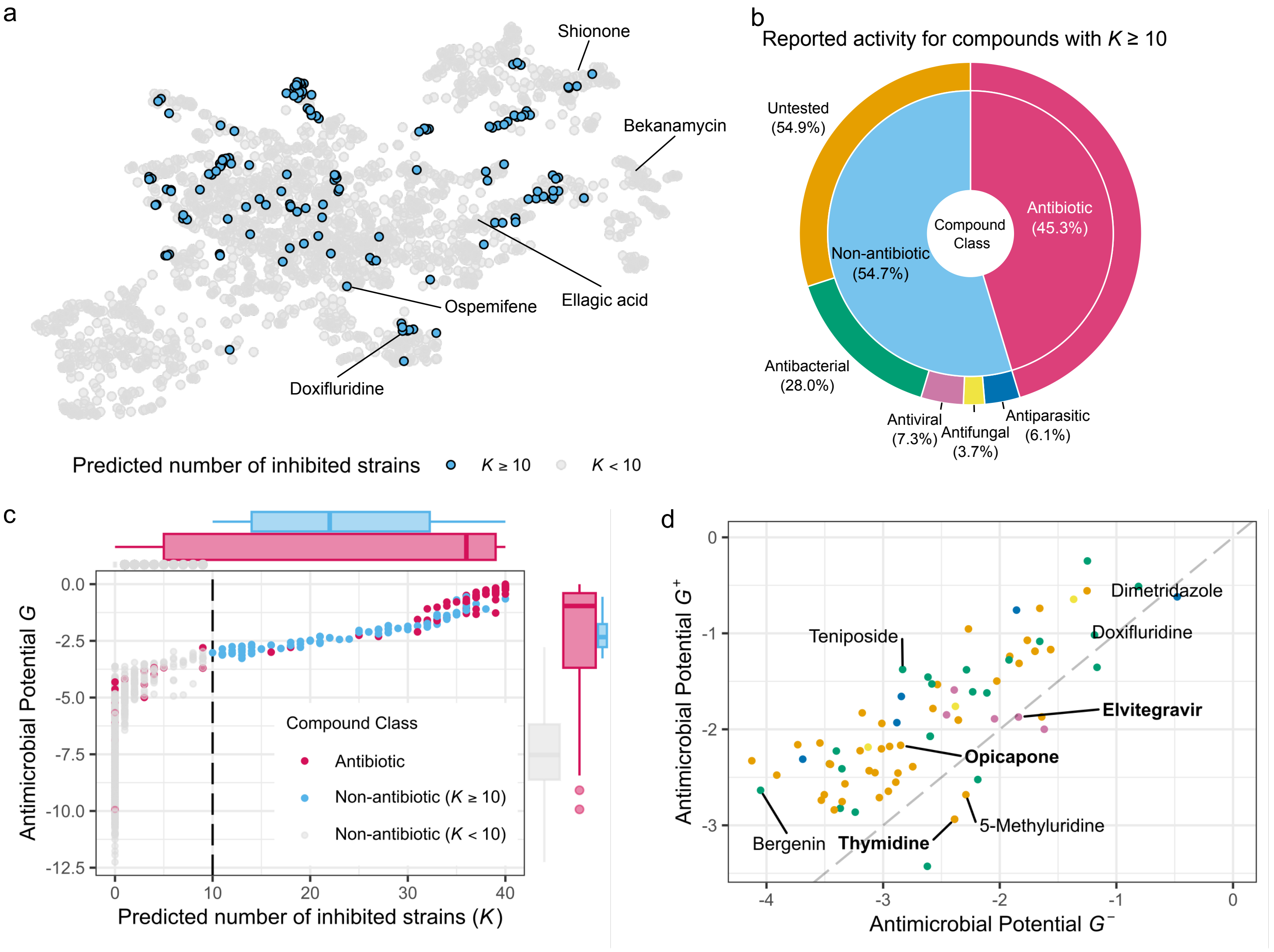

Supplementary Figure 7: Predicting antimicrobial potential in a new chemical library using the ECFP4-based model. **a**. UMAP embedding of the ECFP4 representation of the 2,320 compounds for which predictions are made. Compounds predicted to inhibit 10 or more strains are highlighted in blue. **b**. Literature-reported activity of all compounds predicted to inhibit at least 10 strains. **c**. Ranking of compounds based on Antimicrobial Potential scores. The predicted number of inhibited strains is compared to Antimicrobial Potential G. Known antibiotics are shown in red. Non-antibiotics with $K \geq 10$ ($n = 82$) are shown in blue. **d**. Comparison of the Antimicrobial Potential for Gram-positive (G^+) and Gram-negative strains (G^-) determined for non-antibiotic drugs with predicted broad-spectrum activity. Names shown in bold were selected for experimental validation. The color legend is the same as in panel **b**.

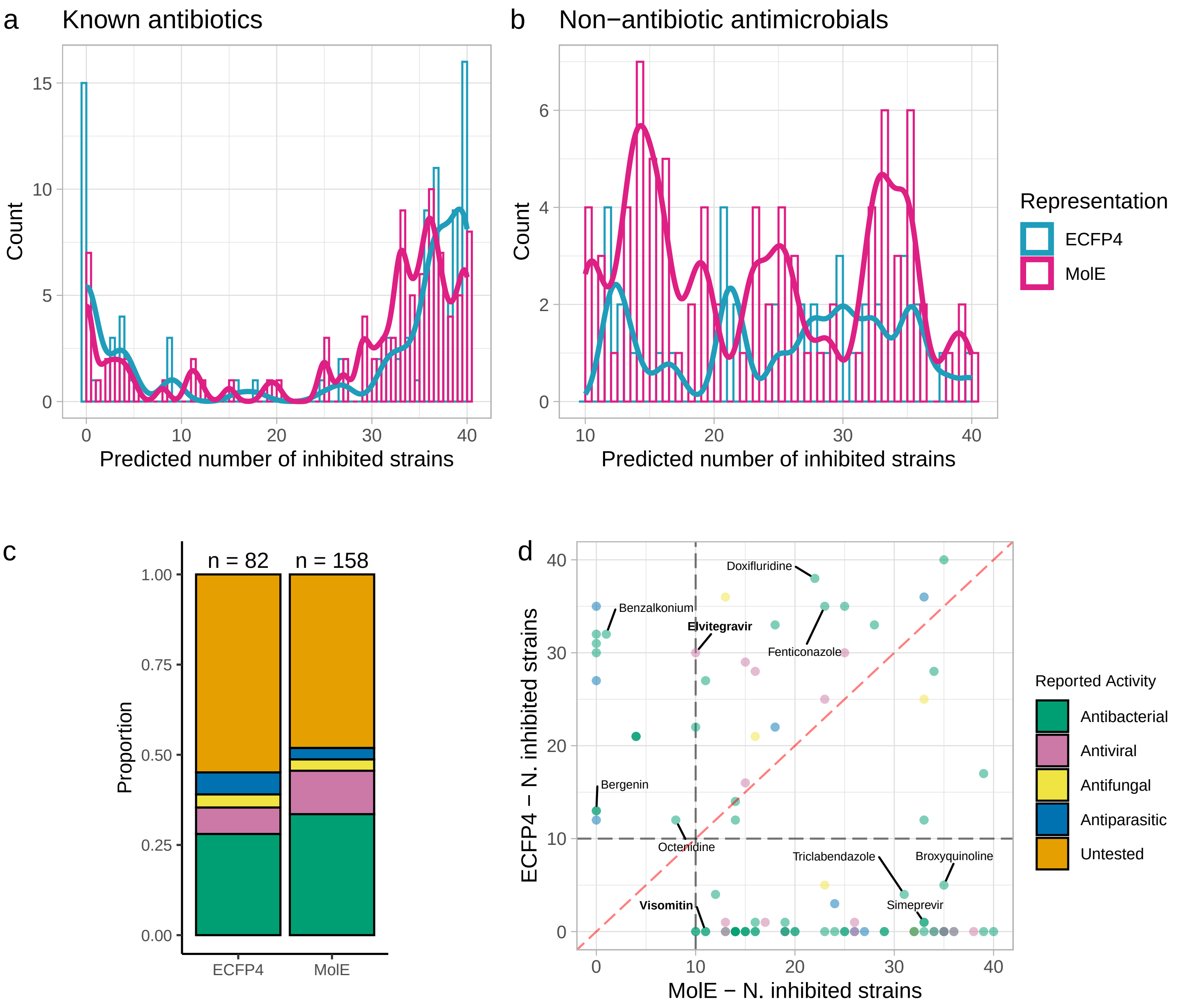

Supplementary Figure 8: Comparison of predictions made on the MedChemExpress library by MolE-XGBoost and ECFP4-XGBoost. **a** Histogram of the predicted number of inhibited strains for known antibiotics ($n = 93$ antibiotics) by ECFP4 and MolE models. **b**. Predicted number of inhibited strains by non-antibiotic compounds with literature-confirmed antimicrobial activity. **c**. Literature search results for non-antibiotic compounds with predicted broad-spectrum activity. **d**. Comparison of the predicted number of inhibited strains for non-antibiotic compounds with confirmed antimicrobial activity. 13 compounds are uniquely recovered by the ECPF4 model, while MolE uniquely recovered 58. Both methods correctly recover the antimicrobial activity of 24 non-antibiotic compounds.

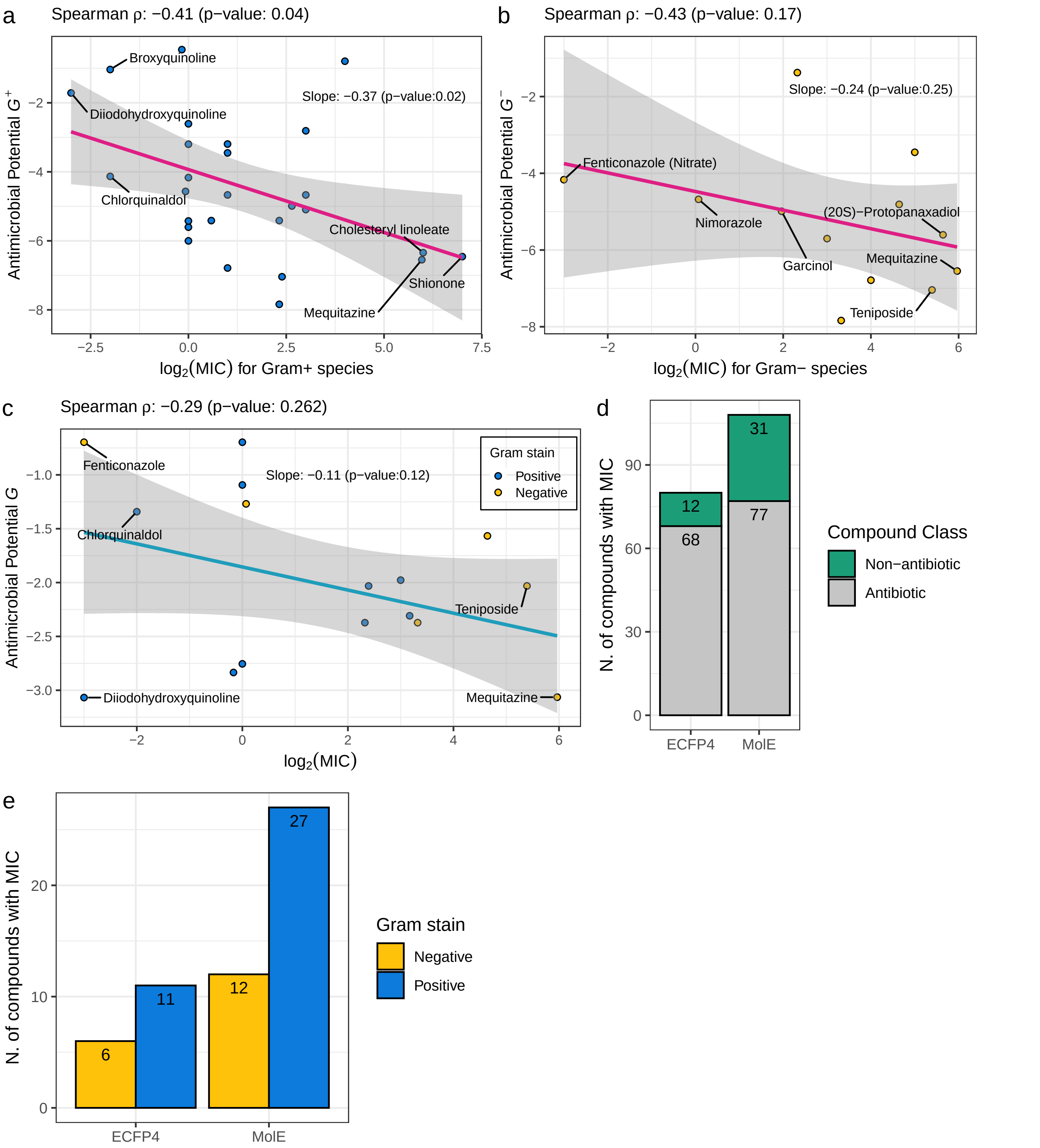

Supplementary Figure 9: AP-scores and reported MIC. **a**. Regression and correlation analysis between the AP-G^+ and the literature-reported $\log_2$ MIC (μg/mL) for 27 non-antibiotic compounds against Gram-positive species. A linear regression fit $\pm$ standard error is shown in pink with grey bands. The slope of the regression is annotated. **b**. Regression and correlation analysis between the AP-G^- and the $\log_2$ MIC for 12 non-antibiotic compounds against Gram-negative species. **c**. Relationship between the AP-score G of the ECFP4 model and the $\log_2$ MIC for the 12 non-antibiotic compounds ($K \geq 10$, according to ECFP4-XGBoost) with a reported MIC ≤ 128 μg/mL against any bacterial species (17 compound-species combinations). **d**. The number of compounds with a reported MIC $\leq 128\mu$g/mL, recovered by MolE and ECFP4. Non-antibiotic and antibiotic compounds are shown. **e**. The number of non-antibiotic compounds with a MIC $\leq 128\mu$g/mL against Gram-positive and Gram-negative species.

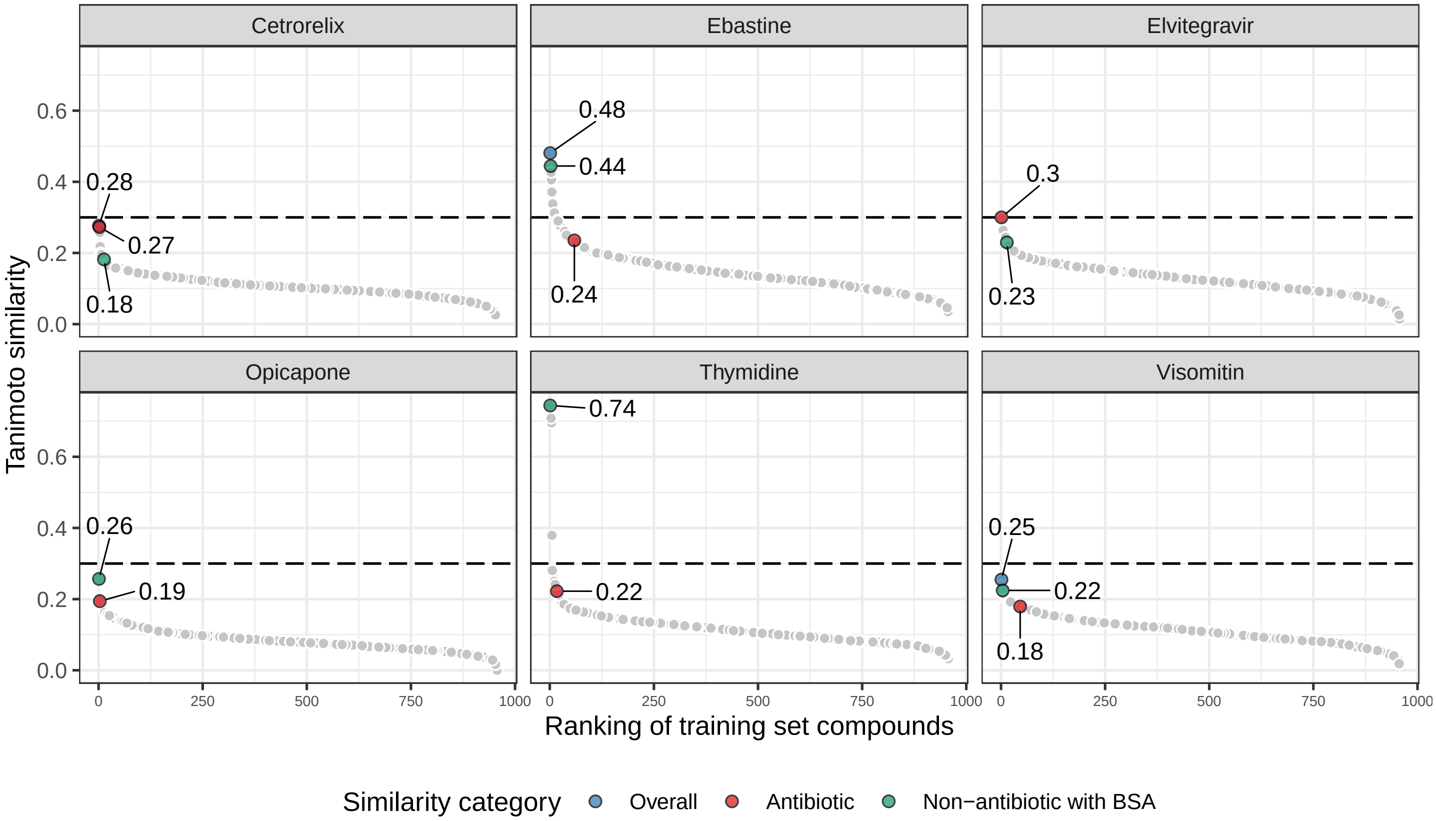

Supplementary Figure 10: Tanimoto similarity between compounds selected for experimental validation and those used for model training is shown. A horizontal line marks a similarity of 0.3. Each compound highlights its most similar antibiotic, broad-spectrum activity (BSA) non-antibiotic, and the most similar training set compound. For Thymidine and Opicapone, the closest training set compound has BSA, while Elvitegravir's closest compound is an antibiotic.

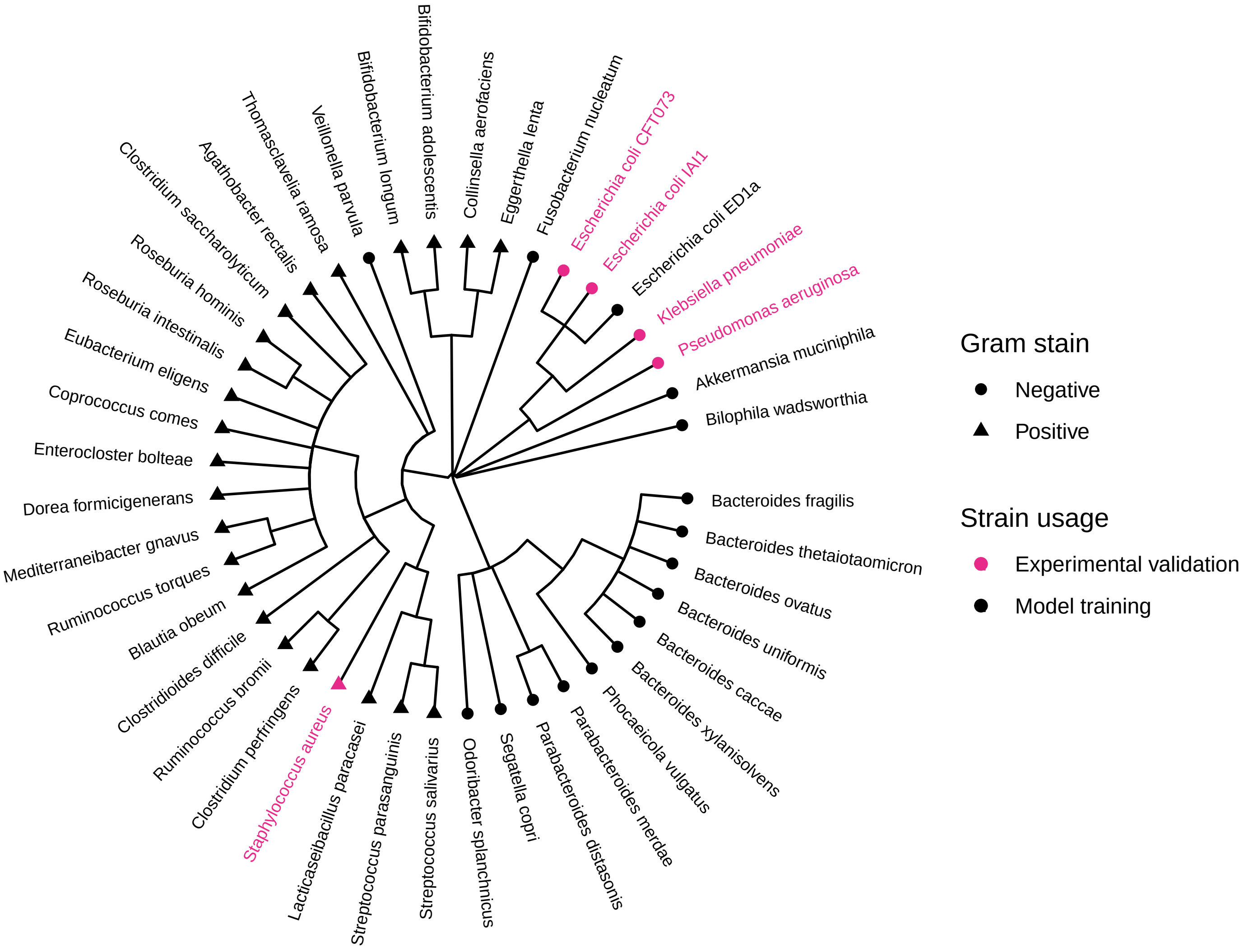

Supplementary Figure 11: Taxonomic tree of the bacterial strains used in this study. Gram stain is shown by tip shape. Strains present in the Maier et.al. dataset and used for model training are shown in black. Strains used for experimental validation are shown in pink.

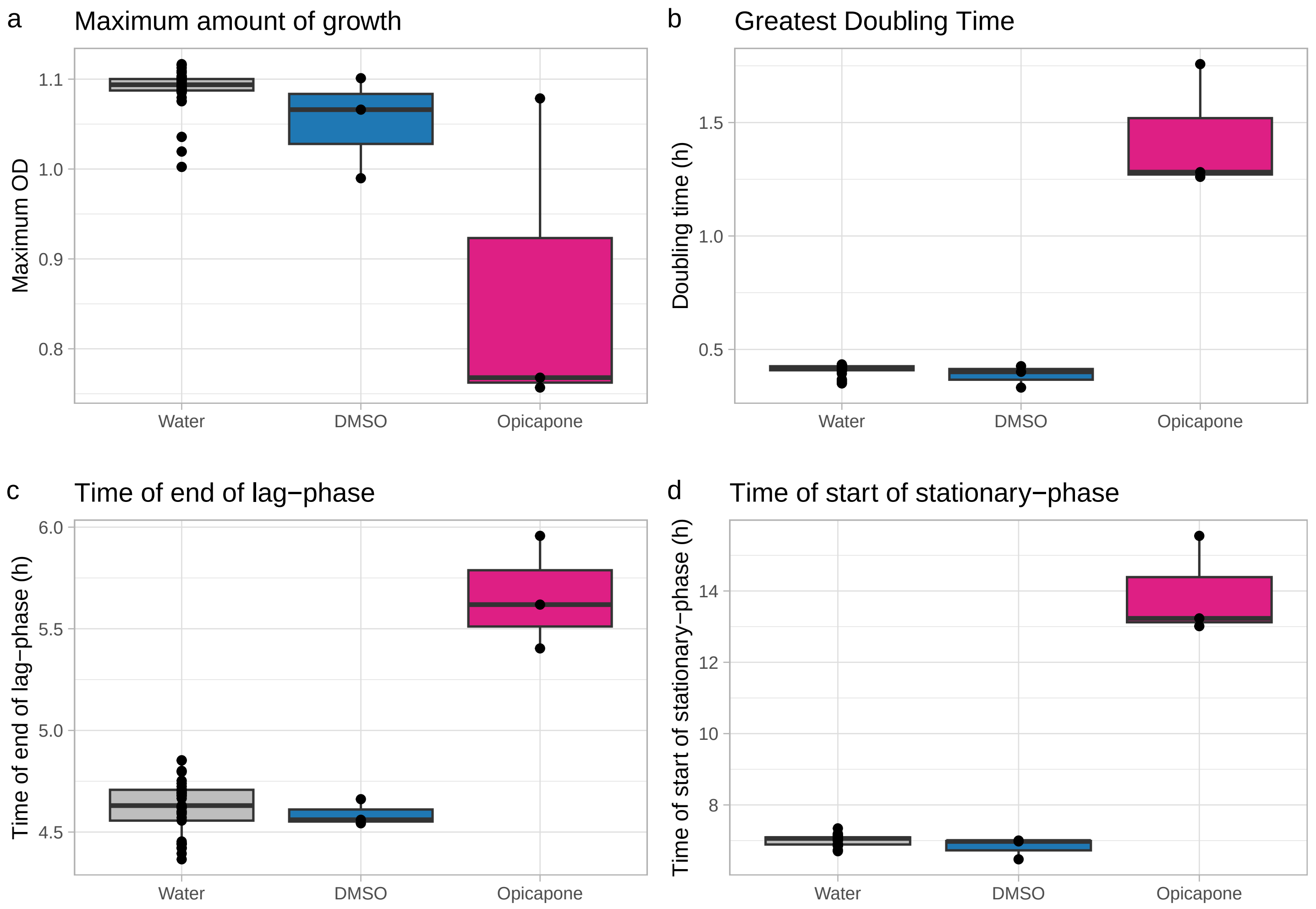

Supplementary Figure 12: Modeled growth parameters for *S. aureus* under Opicapone (16 μg/mL, $n = 3$ biological replicates), DMSO (0.32 μg/mL, $n = 3$ biological replicates) and Water ($n = 33$ spread across 3 biological replicates). **a**. Maximum modeled amount of growth (OD). **b**. Fastest doubling time (hours). **c**. Amount of time (hours) when lag-phase of growth ends. **d**. Amount of time (hours) when the stationary phase is reached.

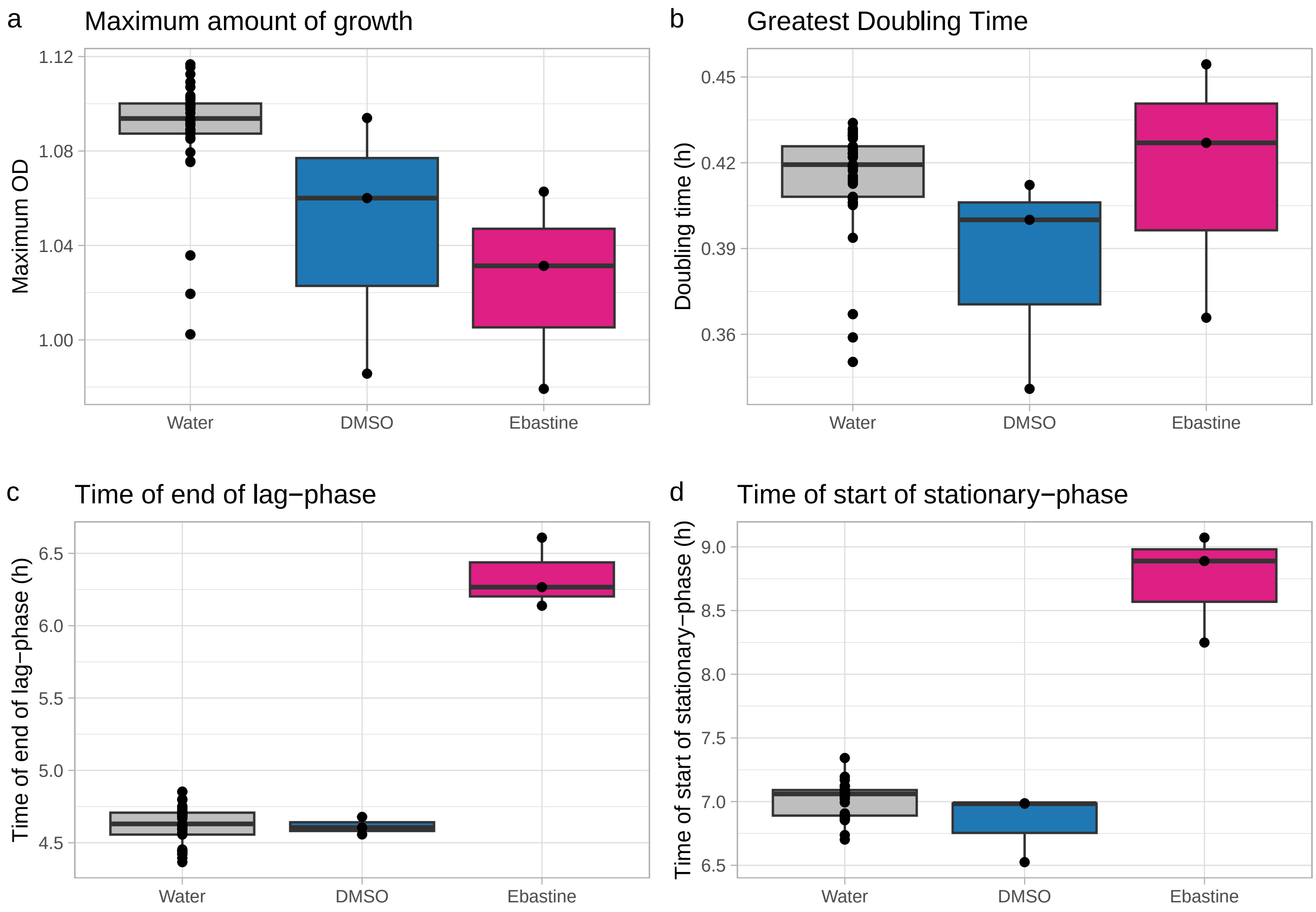

Supplementary Figure 13: Modeled growth parameters for *S. aureus* under Ebastine (16 μg/mL, $n = 3$ biological replicates), DMSO (0.64 μg/mL, $n = 3$ biological replicates), and Water ($n = 33$ spread across 3 biological replicates). **a**. Maximum modeled amount of growth (OD). **b**. Fastest doubling time (hours). **c**. Amount of time (hours) when lag-phase of growth ends. **d**. Amount of time (hours) when the stationary phase is reached.

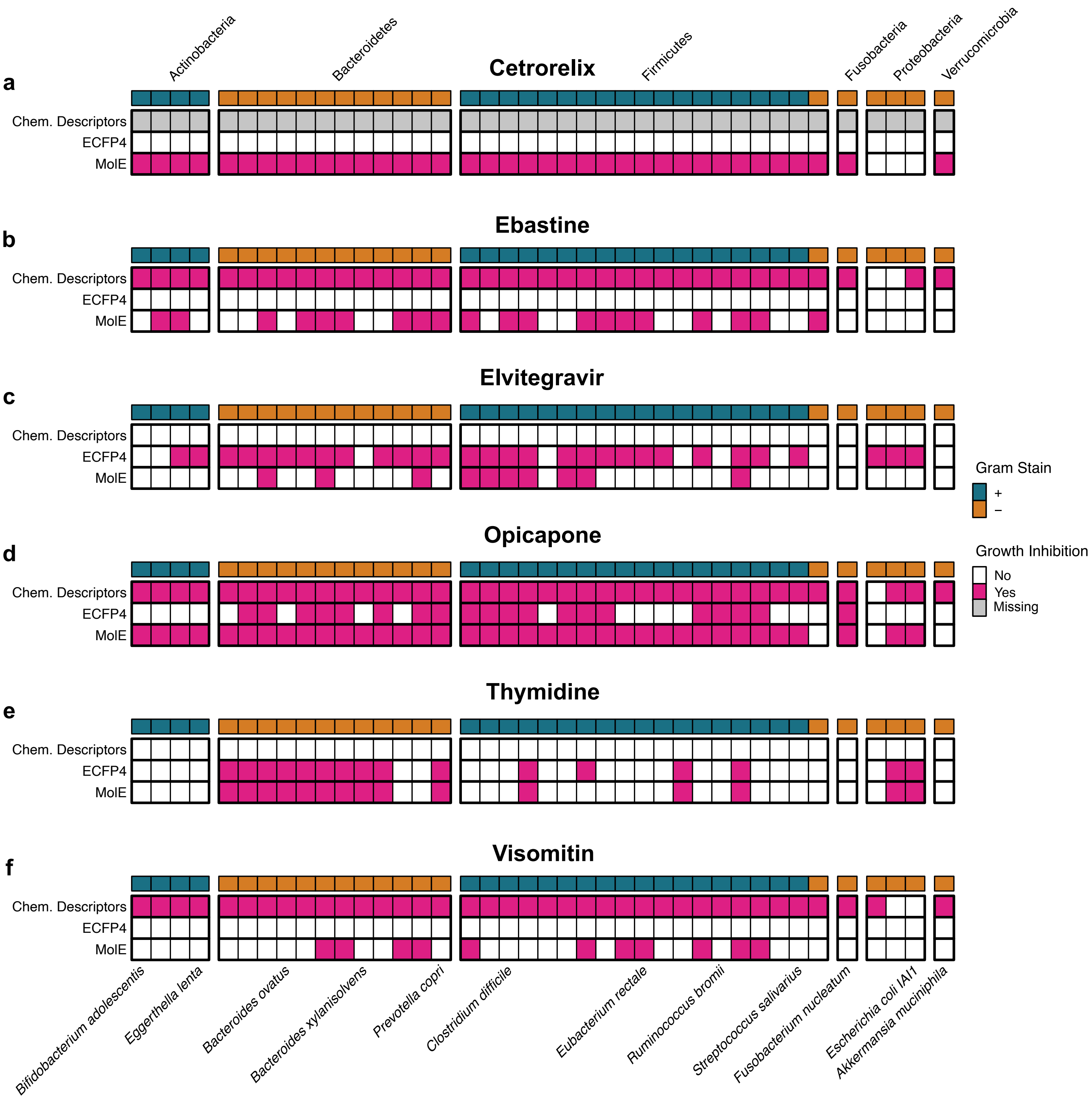

Supplementary Figure 14: Predicted antimicrobial activity of compounds selected for experimental validation. **a**. Cetrorelix. No chemical descriptors were able to be estimated for this compound, therefore no predictions were made. **b**. Ebastine. No antimicrobial activity was predicted when using ECFP4 features. **c**. Elvitegravir. No antimicrobial activity was predicted when using chemical descriptors as features. **d**. Opicapone. All models predict antimicrobial activity. **e**. Thymidine. No antimicrobial activity was predicted when using Chemical Descriptors. **f**. Visomitin. No antimicrobial activity was predicted when using ECFP4 features.

Random Forest Parameters

Name	Possible Values
criterion	"gini", "entropy", "squared_error"*
max_features	"sqrt", "log2", None
n_estimators	100, 300, 500, 700, 1000
class_weight+	"balanced", None

Supplementary Table 4: Random Forest parameters considered during a random search. Some parameters are only considered during *regression or +classification tasks

XGBoost Parameters

Name	Possible Values
max_depth	5, 10, 50, 100
eta	0.3, 0.1, 0.05, 1
n_estimators	30, 100, 300, 500, 1000
subsample	0.3, 0.5, 0.8, 1.0

Supplementary Table 5: XGBoost parameters considered during a random search.

Finetuning parameters

Name	Possible Values
Batch size	32, 100, 512, 800
Learning rate for MLP	5×10^{-4}, 10^{-3}
N layers in MLP head	1, 2
Activation function MLP	"softplus", "relu"
Learning rate for GNN	5 x 10^{-5}, 10^{-4}, 2 x 10^{-4}, 5 x 10^{-4}
Dropout rate	0, 0.1, 0.3, 0.5

Supplementary Table 6: Finetuning parameters considered during a random search.

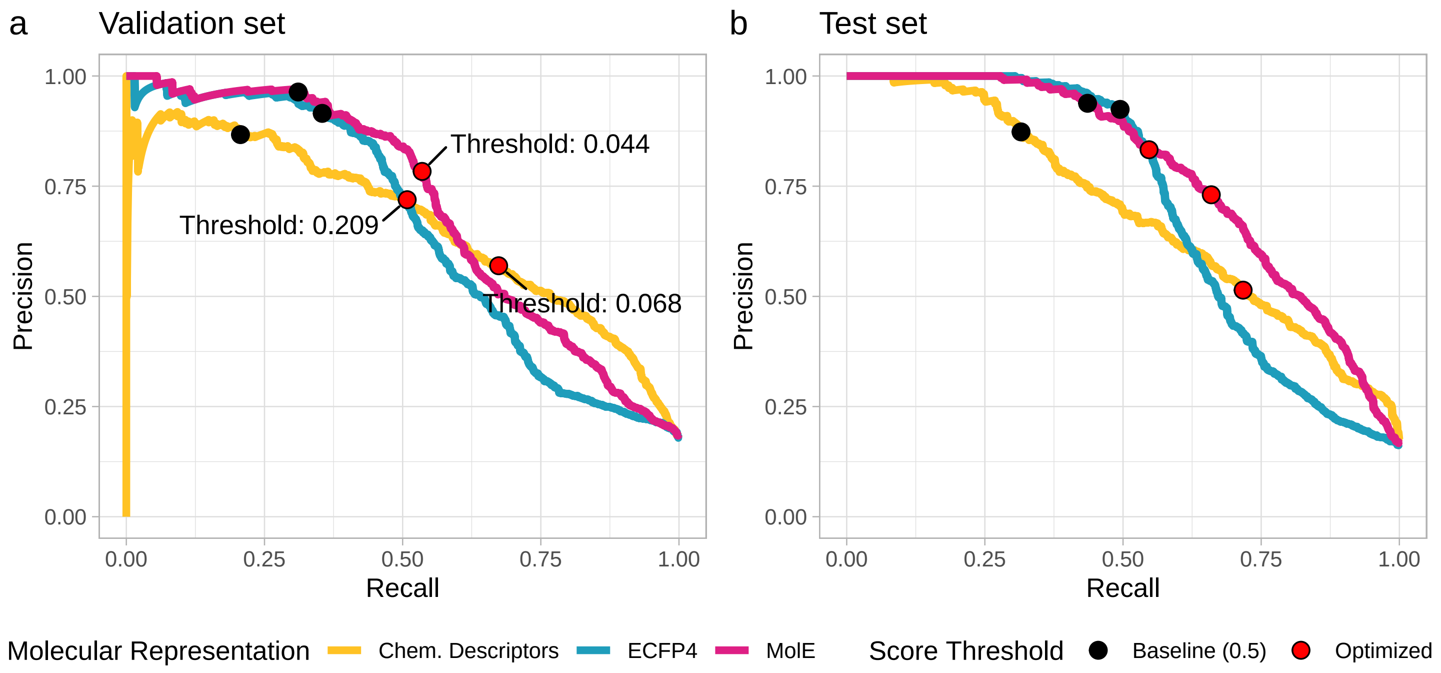

Supplementary Figure 15: Precision-Recall curves for predicting antimicrobial activity in the human gut microbiome. **a** Validation set precision-recall curve. This set was used to determine the score threshold that optimized the trade-off between precision and recall (F1-score). The trade-off with the default threshold (0.5) is shown in black. The optimized trade-off is shown in red. **b**. Test set precison-recall curve. The resulting trade-off between precision and recall using the optimized score determined during validation is also shown.

F1-Score before and after threshold optimization

Set	Threshold	Chem. Descriptors	ECFP4	MolE
Validation	Baseline	0.33	0.51	0.47
	Optimized	0.62	0.60	0.64
Test	Baseline	0.46	0.64	0.60
	Optimized	0.60	0.66	0.69

Supplementary Table 7: . F1-score obtained for predicting antimicrobial activity. Metrics obtained on validation and test sets after binarizing predictions according to baseline (0.5) or optimized score thresholds. Optimized threshold values are determined on the validation set and shown in Supplementary Figure 14

Compounds selected for experimental validation

Name	Summary	Structure
Cetrorelix	A synthetic peptide antagonist of gonadotropin-releasing hormone used to prevent luteinizing hormone surges in women undergoing assisted reproduction therapy	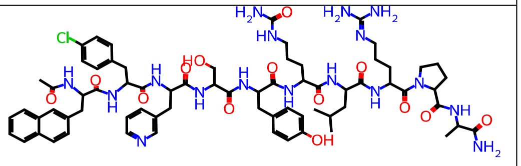
Ebastine	A second generation H1-receptor antagonist useful in the treatment of allergic rhinitis and urticaria.	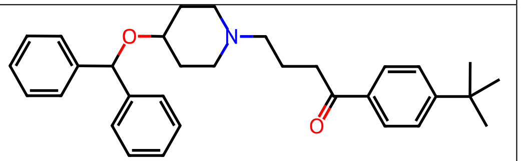
Elvitegravir	An antiretroviral agent used for the treatment of HIV-1 infection.	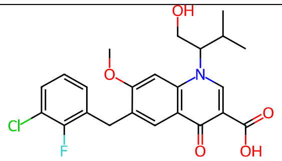
Opicapone	A third-generation catechol-O-methyltransferase inhibitor used as an adjunct treatment for Parkinson's Disease.	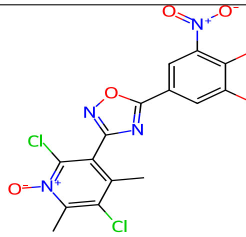
Thymidine	A specific precursor of deoxyribonucleic acid is used as a cell-synchronizing agent.	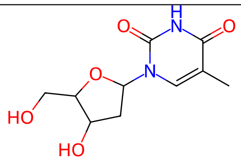
Visomitin	A mitochondrial-targeted antioxidant with a high mitochondrion membrane penetrating ability and potent antioxidant capability.	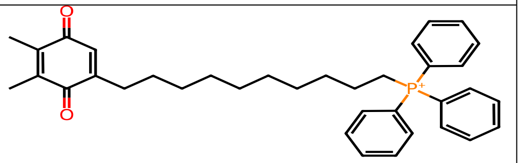

Supplementary Table 8: Compounds selected for experimental validation. Summaries were gathered from DrugBank and MedChemExpress

Bacterial strains used for experimental validation

Strain	Source	Notes
Escherichia coli IAI1	Nassos Typas	Commensal strain
Escherichia coli CFT0731	DSMZ	UPEC model strain (`https://www.dsmz.de/collection/catalogue/details/culture/DSM-103538`)
Pseudomonas aeruginosa PA14	Nassos Typas	Domesticated clinical isolate
Staphylococcus aureus subsp. str. Newman	Nassos Typas	NCTC 8178; ATCC: 13420; `http://www.lgcstandards-atcc.org/Products/All/25904.aspx`
Klebsiella pneumoniae MKP103	Nassos Typas	Lab strain

Supplementary Table 9: . Bacterial strains screened during experimental validation
