# Supplementary material for: Pre-trained molecular representations enable antimicrobial discovery
Source: Nat Commun. 2025 Apr 10;16:3420. doi: 10.1038/s41467-025-58804-4 (PMC11986102; doi:10.1038/s41467-025-58804-4)
Supplement: Supplementary file 2 — Reporting summary [file 41467_2025_58804_MOESM2_ESM.pdf]

nature portfolio

Corresponding author(s): Roberto Olayo-Alarcon and Christian L. Müller

Last updated by author(s): Feb 21, 2025

Reporting Summary

Nature Portfolio wishes to improve the reproducibility of the work that we publish. This form provides structure for consistency and transparency in reporting. For further information on Nature Portfolio policies, see our Editorial Policies and the Editorial Policy Checklist.

Statistics

For all statistical analyses, confirm that the following items are present in the figure legend, table legend, main text, or Methods section.

n/a	Confirmed	
☐	☒	The exact sample size (*n*) for each experimental group/condition, given as a discrete number and unit of measurement
☐	☒	A statement on whether measurements were taken from distinct samples or whether the same sample was measured repeatedly
☐	☒	The statistical test(s) used AND whether they are one- or two-sided<br>*Only common tests should be described solely by name; describe more complex techniques in the Methods section.*
☒	☐	A description of all covariates tested
☒	☐	A description of any assumptions or corrections, such as tests of normality and adjustment for multiple comparisons
☐	☒	A full description of the statistical parameters including central tendency (e.g. means) or other basic estimates (e.g. regression coefficient) AND variation (e.g. standard deviation) or associated estimates of uncertainty (e.g. confidence intervals)
☐	☒	For null hypothesis testing, the test statistic (e.g. *F*, *t*, *r*) with confidence intervals, effect sizes, degrees of freedom and *P* value noted<br>*Give P values as exact values whenever suitable.*
☒	☐	For Bayesian analysis, information on the choice of priors and Markov chain Monte Carlo settings
☒	☐	For hierarchical and complex designs, identification of the appropriate level for tests and full reporting of outcomes
☐	☒	Estimates of effect sizes (e.g. Cohen's *d*, Pearson's *r*), indicating how they were calculated

Our web collection on statistics for biologists contains articles on many of the points above.

Software and code

Policy information about availability of computer code

Data collection	We use the code in the https://github.com/fabkury/atcd repository to gather ATC codes. We also use the pubchempy v1.04 python package in order to gather SMILES from PubChem. OD600 nm measurements were collected using the Synergy H1 plate reade (Aligent).
Data analysis	All custom code used for data analysis, predictions, and model training are made publicly available in our GitHub repositories https://github.com/rolayoalarcon/MolE and https://github.com/rolayoalarcon/mole_antimicrobial_potential. All python code is implemented in Python 3.7 and R code is implemented in R 4.3.1. The MolE pre-training framework was implemented using the pytorch-geometric 1.6.3 framework with Python 3.7. We use the RandomForestClassifier and RandomForestRegressor implementation available in scikit-learn 1.0.2 and the XGBClassifier and XGBRegressor objects from xgboost 1.6.2. The scikit-learn 1.0.2 module is also used when computing ROC-AUC, PR-AUC, and F1 score metrics. The R 4.3.1 language was used for its ggplot2 3.4.2 for plotting, Sicegar 0.2.4 packages. ECFP4, chemical descriptors, and general SMILES processing were done with the rdkit 2020.09.1.0 package.

For manuscripts utilizing custom algorithms or software that are central to the research but not yet described in published literature, software must be made available to editors and reviewers. We strongly encourage code deposition in a community repository (e.g. GitHub). See the Nature Portfolio guidelines for submitting code & software for further information.

Data

Policy information about availability of data

All manuscripts must include a data availability statement. This statement should provide the following information, where applicable:

- Accession codes, unique identifiers, or web links for publicly available datasets
- A description of any restrictions on data availability
- For clinical datasets or third party data, please ensure that the statement adheres to our policy

The unlabeled chemical structures used for pre-training were gathered from the MolCLR GitHub repository https://github.com/yuyangw/MolCLR. The adjusted p-value table from Maier. L., et.al. 2018, was used to train models to predict antimicrobial activity and was gathered from the respective publication.
The chemical library from MedChemExpress can be found in our GitHub repository https://github.com/rolayoalarcon/mole_antimicrobial_potential (doi: 10.5281/zenodo.15089370).
Results for predicting antimicrobial activity,
and the data from experimental validation are available at
https://github.com/rolayoalarcon/mole_antimicrobial_potential (doi: 10.5281/zenodo.15089370).
All data is publicly available and can be accessed without restrictions.
Source data are provided with this paper.

Research involving human participants, their data, or biological material

Policy information about studies with human participants or human data. See also policy information about sex, gender (identity/presentation), and sexual orientation and race, ethnicity and racism.

Reporting on sex and gender	NA
Reporting on race, ethnicity, or other socially relevant groupings	NA
Population characteristics	NA
Recruitment	NA
Ethics oversight	NA

Note that full information on the approval of the study protocol must also be provided in the manuscript.

Field-specific reporting

Please select the one below that is the best fit for your research. If you are not sure, read the appropriate sections before making your selection.

☒ Life sciences ☐ Behavioural & social sciences ☐ Ecological, evolutionary & environmental sciences

For a reference copy of the document with all sections, see nature.com/documents/nr-reporting-summary-flat.pdf

Life sciences study design

All studies must disclose on these points even when the disclosure is negative.

Sample size	No sample size calculations were performed for this study. Three biological replicates are performed as is convention. All experiments were performed in the same experimental conditions, with the only varying covariate being the treatment applied.
Data exclusions	We did not consider 4 growth curves gathered for S.aureus in the presence of Water, due to evident overgrowth (max OD > 1.3). We had previously determined that compounds with overgrowth should not be considered so as to not overestimate microbial growth in control conditions. The code used to filter these examples is in our GitHub repository https://github.com/rolayoalarcon/mole_antimicrobial_potential
Replication	Microbial growth experiments were performed in biological triplicate for each compound and DMSO for each concentration, with the exception of growth in response to Water for which 11 growth curves were gathered for each of the three biological replicates (33 growth curves in total). All attempts at replication were successful.
Randomization	In vitro experiments were performed, where treatment placement was done with prior knowledge. Randomization not necessary/not applicable.
Blinding	In vitro experiments were performed, where treatment placement was done with prior knowledge. Blinding not necessary/not applicable.

Reporting for specific materials, systems and methods

We require information from authors about some types of materials, experimental systems and methods used in many studies. Here, indicate whether each material, system or method listed is relevant to your study. If you are not sure if a list item applies to your research, read the appropriate section before selecting a response.

Materials & experimental systems

n/a	Involved in the study
☒	☐ Antibodies
☒	☐ Eukaryotic cell lines
☒	☐ Palaeontology and archaeology
☒	☐ Animals and other organisms
☒	☐ Clinical data
☒	☐ Dual use research of concern
☒	☐ Plants

Methods

n/a	Involved in the study
☒	☐ ChIP-seq
☒	☐ Flow cytometry
☒	☐ MRI-based neuroimaging

Plants

Seed stocks	NA
Novel plant genotypes	NA
Authentication	NA
